# Honeybee Venom Proteome Profile of Queens and Winter Bees as Determined by a Mass Spectrometric Approach

**DOI:** 10.3390/toxins7114468

**Published:** 2015-10-30

**Authors:** Ellen L. Danneels, Matthias Van Vaerenbergh, Griet Debyser, Bart Devreese, Dirk C. de Graaf

**Affiliations:** 1 Laboratory of Molecular Entomology and Bee Pathology, Ghent University, Krijgslaan 281 S2, B-9000 Ghent, Belgium; E-Mail: dirk.degraaf@ugent.be; 2 Laboratory of Protein Biochemistry and Biomolecular Engineering, Ghent University, K.L. Ledeganckstraat 35, B-9000 Ghent, Belgium; E-Mails: grietdebyser@hotmail.com (G.D.); bart.devreese@ugent.be (B.D.)

**Keywords:** honeybee, venom, mass spectrometry, queen, seasonal variation, caste differentiation, vitellogenin

## Abstract

Venoms of invertebrates contain an enormous diversity of proteins, peptides, and other classes of substances. Insect venoms are characterized by a large interspecific variation resulting in extended lists of venom compounds. The venom composition of several hymenopterans also shows different intraspecific variation. For instance, venom from different honeybee castes, more specifically queens and workers, shows quantitative and qualitative variation, while the environment, like seasonal changes, also proves to be an important factor. The present study aimed at an in-depth analysis of the intraspecific variation in the honeybee venom proteome. In summer workers, the recent list of venom proteins resulted from merging combinatorial peptide ligand library sample pretreatment and targeted tandem mass spectrometry realized with a Fourier transform ion cyclotron resonance mass spectrometer (FT-ICR MS/MS). Now, the same technique was used to determine the venom proteome of queens and winter bees, enabling us to compare it with that of summer bees. In total, 34 putative venom toxins were found, of which two were never described in honeybee venoms before. Venom from winter workers did not contain toxins that were not present in queens or summer workers, while winter worker venom lacked the allergen Api m 12, also known as vitellogenin. Venom from queen bees, on the other hand, was lacking six of the 34 venom toxins compared to worker bees, while it contained two new venom toxins, in particularly serine proteinase stubble and antithrombin-III. Although people are hardly stung by honeybees during winter or by queen bees, these newly identified toxins should be taken into account in the characterization of a putative allergic response against *Apis mellifera* stings.

## 1. Introduction

Hymenopteran venoms are a complex mixture of substances, mainly composed of proteins and peptides, but also containing alkaloids, amines, and other small molecules [1]. The venom of a single species can contain hundreds to several thousands of active compounds, while the overall venom content variation between species is enormous. Global comparison of venom electrophoretic profiles of 25 different hymenopteran species revealed that the protein patterns strongly differed from one species to another [2]. Even when comparing the venoms of two *Apis mellifera* subspecies, *A. mellifera carnica* and *A. mellifera ligustica*, to the venom of the Africanized honeybee hybrid, differences in the presence and abundance of venom compounds could be noticed [3]. 

In several insects, including ants, bees, and wasps, eusociality is observed and studied, which is typically characterized by a division of labor into reproductive and non-reproductive groups [4]. This creates specialized behavioral groups within an animal society that are called castes. The colony of the fire ant *Solenopsis invicta*, for instance, can contain reproductive queens, un-reproductive queens, workers, and drones. The three first mentioned female castes differed both qualitatively and quantitatively in their venom composition, in particularly in the venom alkaloids [5]. This variability in venom composition between queen and worker ants is not so surprising when looking at the functionality of the venom. While workers inject venom directly into other animals for defense and predation, or spray it throughout the nest environment, presumably for protection against microbial pathogens, queens apply it over eggs as they are laid, either for fungal protection or to advertise their presence and fertility [6]. This difference in venom functionality is even more clear in honeybees, where workers use their venom for defense of the colony against predators and intruders, while queens use it only for offense—the stinging and killing of rival queens in the colony [7]. Accordingly, the venom from young queens, who typically use their venom against rivals, is half as lethal to mice as that of their conspecific workers [7]. When looking at one of the major honeybee venom components, hyaluronidase, its concentration and activity is considerably higher in workers than in queens, which is probably associated with the actions of bee sting against mammals [8]. When a mammal is stung, the venom must spread through the tightly packed connective tissue in order to cause pain. When a queen stings her rival queen, the venom is mostly delivered straight into the hemocoel and spread immediately with the hemolymph flow to its site of action. This route involves minimal intercellular penetration and therefore does not require high levels of hyaluronidase activity. Next to hyaluronidase, workers were also previously proven to contain 50 times more secapin [9], less histamine, more phospholipase A_2_, and half of the amount of melittin [7] compared to queen honeybees. 

Also, the age of the honeybee can define its venom content. The start of venom gland activity differs between the honeybee workers and queen, due to the fact that both castes need a functional venom at a different time in their life [10]. Queens use the venom upon emergence to fight with other queens. They can live up to five years, but by the time they reach the age of one to two years, their venom has become inactive [7]. In contrast, workers use the venom when performing tasks outside the hive, which starts around the 20th day of adult life. The venom glands of both castes have only one secretory cycle, which starts at the end of pupation in queens and just after emergence in workers. In workers, the highest secretory activity of the venom glands is reached around the 16th day. Biochemical studies revealed that the activity of some hydrolytic enzymes from the venom glands of *A. mellifera* was higher in workers of 14 days of age than in those of 40 days [11]. Temporal changes in melittin, histamine, and hyaluronidase have previously been reported in honeybee workers and queens [7,8]. 

With regard to this intraspecific venom variation, the environment has also proven to be an important factor. For instance, the venom of the giant ant *Dinoponera quadriceps* collected in four different areas of Brazil showed major differences in composition; venom collected in the closest areas seemed more similar than the ones collected in distant regions [12]. Previously, the presence of alkaloids in venom from the fire ant *Solenopsis* species was mentioned. The concentration of these alkaloids and the venom volume was not only proven to be higher for soldiers (major workers) than for workers (minor workers) representing caste differences [13], but also showed seasonal variation. More specifically, the ratio of cis C11 to trans C11 alkaloids in the venom of minor workers was the highest in spring and the lowest in winter [14]. 

When studying the intraspecific diversity of melittin and phospholipase A_2_ in venom from honeybees, Ferreira Junior and collaborators could associate the variation of the venom composition with climatic and seasonal factors [15]. Seasonal variation was also noticed for the antigen 5-like gene that is expressed by the venom gland tissue of winter bees but not of summer bees [16]. Winter worker bees differ a lot from summer workers since they rarely leave the hive for many months. They are reared in late summer and autumn, fit to survive the cold season, and form the winter cluster without brood rearing. Instead of becoming foragers, the young winter workers enter the diutinus stage and live 22 to 24 weeks, while summer workers only live four to six weeks. During winter in the temperate zone, the workers face different predators and intruders than during the summer: for example, mice often try to take shelter in a honeybee hive during the winter months, while wasps are not active during winter months. This means that the function together with the composition of the venom possibly differs from summer worker venom. Next to that, the repertoire of allergens known today is nearly completely defined by the allergic reaction of people that are stung during summer. 

Next to environmental influences, intraspecific variation in hymenopteran venoms can be as extreme as showing differences between individuals from the same population with the same age. This was recently investigated for the parasitoid wasp *Leptopilina boulardi* by electrophoretic profiles of individual venoms showing both qualitative (presence/absence) and quantitative (intensity of specific bands) inter-individual variation [17]. 

The venom proteome of the honeybee *A. mellifera* was recently investigated by integrating a combinatorial peptide ligand library approach with nanoLC FT-ICR MS/MS [18], resulting in 102 venom proteins and peptides, of which 33 were categorized as putative venom toxins. While this in-depth analysis was performed on venom from worker bees collected during the summer, the present study aimed to examine possible caste and/or seasonal variation in the venom composition of *A. mellifera carnica*. Therefore, we used similar techniques on venom collected from queens and winter workers and compared the data to previous results.

## 2. Materials and Methods

### 2.1. Venom Collection

Mid-February 2011, adult worker honeybees (*A. mellifera carnica*) were collected at the hive entrance. Mid-June 2012, queen honeybees were reared by routine apicultural techniques. Queen venom was collected within 24 hours after emergence. Pure venom was collected by manual milking, as previously described [19]. Venom of 150 winter workers was pooled to a protein concentration of 71.26 mg/mL, as determined by Bradford protein assay (Thermo Scientific Pierce, Hudson, NH, USA). Venom of 37 queen honeybees was pooled to a protein concentration of 49.175 mg/mL. 

### 2.2. Mass Spectrometric Analysis

Unless otherwise indicated, all experiments were conducted as described in a preceding honeybee venom proteome study [16]. The dynamic range of protein concentrations in the venom samples was compressed by a combinatorial peptide ligand library (CPLL) approach (ProteoMiner protein enrichment small-capacity kit, Bio-Rad Laboratories, Hercules, CA, USA). Approximately 200 μg and 100 μg of the CPLL flow-through and eluted protein fractions of winter worker bee venom respectively, were separated on a 10% Tris-glycine-SDS-PAGE gel. In addition, 50 μg of CPLL flow-through proteins and 100 μg of CPLL elution proteins were separated on a 16.5% Tris-tricine-SDS-PAGE gel. Approximately 100 μg and 50 μg of the CPLL flow-through and eluted protein fractions of queen bee venom respectively, were separated on a 10% Tris-glycine-SDS-PAGE gel. In addition, 25 μg of CPLL flow-through proteins and 50 μg of CPLL elution proteins were separated on a 16.5% Tris-tricine-SDS-PAGE gel. After Coomassie staining, proteins were in-gel reduced and alkylated. All flow-through and elution bands larger than 40 kDa were cut out of the 10% glycine gel, while those smaller than 40 kDa were cut out of the 16.5% tricine gel. Also, gel parts without any visible protein bands were excised and analyzed. After in-gel tryptic digestion, tryptic peptides were extracted from the gel and analyzed by LC-ESI-LTQ-FT-ICR MS/MS. MS/MS data were searched using the Mascot v2.3 database search engine (Matrix Science, London, UK) against the Amel4.5 NCBI Refseq (available at ftp://ftp.ncbi.nih.gov/genomes/Apis_mellifera/protein/; database contains 21,772 sequences and 14,426,513 residuals) and Augustus9 (available at ftp://ftp.hgsc.bcm.edu/Amellifera/Amel_4.5GenePredictions/Augustus/; database contains 11,560 sequences and 7,698,817 residuals) protein prediction databases. An automatic decoy database search was performed to enable false discovery rate (FDR) determination. The significance threshold was adapted to 0.001 to reach a FDR < 1% for the identity threshold of both database searches. Searches were executed with carbamidomethylation of cysteines as a fixed modification and oxidation of methionines as a variable modification. One tryptic miscleavage was permitted and peptide mass tolerance and MS/MS tolerance were set to 10 ppm and 0.3 Da, respectively. Precursor peptide charge states were set to 2+ and 3+. 

### 2.3. Criteria for Positive Identifications

We defined positive protein identifications as queries detected by at least two unique, bold, and red (significant and top ranking) peptides from the Mascot output with an ion score ≥ 30. In addition, the discovery of small peptides was enabled by allowing queries with a sequence coverage higher than 10% due to the detection of only one, bold and red (significant and to ranking) peptide with an ion score ≥ 30. All protein identifications were merged in one list and all double identifiers were removed. 

### 2.4. Sequence Analysis

Subsequently, a bioinformatics analysis of identified compounds was executed. The presence of secretion signal peptides, protein domains, protease and protease inhibitor families, and GO-terms, and the existence of similar proteins in exosomes and venoms of other species, were determined as previously described [16]. 

## 3. Results and Discussion

### 3.1. Identification and Categorization of Venom Proteins

**Figure 1:**
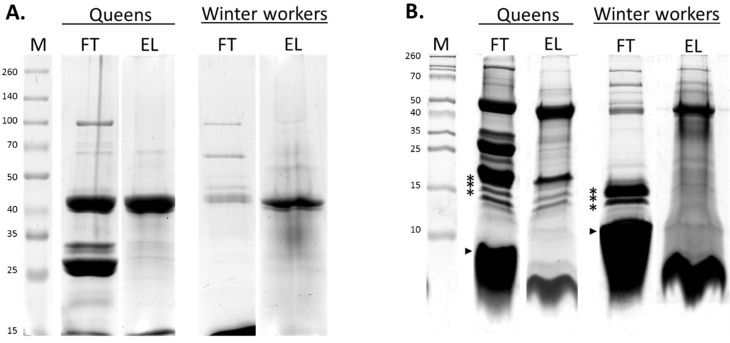
Electrophoretic separation of 2 CPLL-treated honeybee venom samples. On the left, venom from queen honeybees was collected; on the right, venom from winter worker honeybees was collected. CPLL flow-through (=FT) and elution (=EL) samples are separated on a 10% Tris-glycine-SDS-PAGE gel (**A**) and a 16.5% Tris-tricine-SDS-PAGE gel (**B**). Molecular weight regions that are known to contain high amounts of PLA2 (*) and melittin (►) are indicated. Molecular weights (in kDa) of the markers are indicated in the figure: (**A**) PageRuler Prestained Protein Ladder; (**B**) Spectra Multicolor Low Range Protein Ladder.

The in-depth proteomic analysis of the venom from winter workers of *A. mellifera* revealed 656 unique tryptic peptides (see Supplementary Tables S1 and S2), providing biological evidence for 88 venom proteins and peptides. Queen venom, on the other hand, revealed 521 unique tryptic peptides (see Supplementary Tables S1 and S2), providing biological evidence for 76 venom proteins and peptides. In a preceding study, honeybee venom compounds were categorized into the groups of putative toxins and venom trace molecules by prediction of their biological function and subcellular location [16]. Since less abundant compounds were enriched by the CPLL pretreatment (Figure 1), a subgroup of identified proteins was found that probably only have a local function in the venom duct or reservoir or are released by leakage of the gland tissue. This group of venom trace molecules is fully listed in Supplementary Table S3. At present, no function could be attributed to 16 compounds. They were categorized separately (Supplementary Table S4) because they lacked functional domains and/or similar annotated sequences. In contrast to venom trace molecules, venom toxins are typically highly abundant and are actively secreted by the venom glands to contribute to the venom defense or social immunity function. In total, 34 toxins are listed in Table 1, complemented by the compounds that are also secreted but show no clear toxic venom function and therefore are categorized under venom trace molecules. Of these 34 putative venom toxins, two had not been discovered in honeybee venom before (serine proteinase stubble and antithrombin-III). Despite the identification of new venom toxins, some of the previously reported honeybee venom compounds were missing from our list. The antigen 5-like wasp venom paralog was previously proven to show seasonal variation since it could be expressed by venom gland tissue of winter bees but not of summer bees [16]. However, this antigen 5-like venom protein could not be detected by our FT-ICR MS studies in any of the three (summer worker, winter worker, queen) venom samples, which could be due to venom sample variation or technological variation (liquid *versus* gel-based proteomics). Other honeybee venom compounds that have been described long ago (like cardiopep and minimine) together with some small peptides (like tertiapin and apidaecin) were lacking in the list of toxins found by FTMS in summer worker venom [18]. Yet, the present study also failed to identify these compounds using the same techniques on venom from honeybee queen and winter workers. However, vitellogenin, one of the venom compounds that was lacking in the MS study on summer worker venom, could now be detected in venom from *A. mellifera* queens. 

### 3.2. Comparison of the Venom Composition between Summer and Winter Worker Honeybees

The current study revealed no venom toxins that were uniquely present in winter worker honeybees. On the other hand, three venom toxins previously found in summer workers by combining CPLL sample pretreatment and nanoLC FT-ICR MS/MS, were lacking in winter worker venom analyzed by similar techniques. These toxins are group XV phospholipase A_2_, putative trypsin, and c-type lectin. 

**Table 1:** All discovered putative toxins, together with a selected group of venom trace molecules, are classified according to their function. GenBank accession numbers (Acc. N°) and allergen names (Allergen) are shown. The presence of a secretion signal peptide (derived by SignalP) is marked with “x”. Also, proteins that exist in the exosome protein database ExoCarta are indicated with “x” (EX). The presence of the listed proteins in the preceding honeybee venom study on summer worker venom is marked with “x” (Summer worker), together with their presence in one of the current venom studies on winter worker and queen honeybees. When the protein was not found in our study, but could be detected by another proteome study, the x is between brackets. The results of standalone blasts against a venom sequence database, which reveals the existence of similar venom proteins and venom gland transcripts (Evidence) of other species (Species), are shown. Type of evidence: P = venom protein; T = venom gland transcript; EA = enzymatic activity; U = unknown.

Name	Acc. N°	Allergen	SignalP	EX	Summer Worker	Winter Worker	Queen	Similar Venom Compound		
								Species	Evidence	Ref.
***A. Putative* toxins**										
**Esterases**										
Phospholipase A2-1	gi|58585172	Api m 1	x	-	x	x	x	-	-	-
Phospholipase A2-2	gi|110758297	-	x	-	x	x	x	-	-	-
Group XV phospholipase A2	gi|328791555	-	x	-	x	-	-	*Ophiophagus hannah*	P+T	[20]
Acid phosphatase	gi|301601654	Api m 3	x	x	x	x	x	*Nasonia vitripennis*	P	[21]
Acid phosphatase 2	gi|571531089	-	x	x	x	x	x	*Pteromalus puparum*	T+EA	[22]
5′-Nucleotidase	gi|66523706	-	x	x	x	x		*Gloydius blomhoffi*	P	[23]
Carboxylesterase	gi|187281550	Api m 8	x	x	x	x		*Nasonia vitripennis*	P	[21]
**Proteases and peptidases**										
CLIP serine protease	gi|571522677	-	x	-	x	x	x	*Bombus ignitus*	P	[24]
CUB serine protease 1	gi|58585116	Api m 7	x	-	x	x	x	*Nasonia vitripennis*	T	[21]
CUB serine protease 2	gi|48101366	-	x	-	x	x	x	*Nasonia vitripennis*	T	[21]
Putative trypsin	au9.g8903.t1	-	x	-	x	-	x	*Nasonia vitripennis*	P	[21]
Serine protease snake	gi|328783264	-	x	-	x	x	-	*Ophiophagus hannah*	P+T	[20]
Dipeptidyl peptidase IV	gi|187281543	Api m 5	x	x	x	x	x	*Vespula vulgaris*	P	[25]
Serine carboxypeptidase	gi|226533687	Api m 9	x	x	x	x	x	*Croatlus adamanteus*	T	[26]
Prolylcarboxypeptidase	gi|328778095	-	x	x	x	x	x	*Ophiophagus hannah*	P+T	[20]
Metalloprotease	gi|571501445	-	x	x	x	x	x	*Eulophus pennicornis*		[27]
Serine proteinase stubble	au9.g5504.t1	-	x	-	-	-	x	*Crotalus adamanteus*	T	[26]
**Protease inhibitors**										
Api m 6	gi|94400907	Api m 6	x		x	x	x	-	-	-
Serpin 1	gi|328793022	-	x	x	x	x	x	*Ophiophagus hannah*	P+T	[20]
Serpin 2	gi|328791596	-	x	x	x	x	x	*Tityus bahiensis*	T	[28]
Serpin 3	gi|328780925	-	x	x	x	x		*Tityus bahiensis*	T	[28]
Antithrombin-III	gi|571552510	-	x	x	-	-	x	*Tityus bahiensis*	T	[28]
**Carbohydrate metabolism**										
Hyaluronidase	gi|58585182	Api m 2	x	-	x	x	x	*Apis cerana*	T	[29]
N-sulfoglucosamine sulfohydrolase	gi|328793712	-	x	-	x	x	x	*Boiga irregularis*	T	[30]
Endochitinase	gi|66511507	-	x	-	x	x	x	*Nasonia vitripennis*	T	[21]
**Growth factors**										
Platelet-derived growth factor	gi|571515288	-	x	x	x	x	x	*Echis coloratus*	T	[31]
Imaginal disc growth factor 4	gi|571545715	-	x	-	x	x	x	*Chelonus inanitus*	P	[32]
**Major royal jelly proteins**										
MRJP8	gi|58585070	Api m 11.0101	x	-	x	x	x	*Chelonus inanitus*	P	[32]
MRJP9	gi|67010041	Api m 11.0201	x	-	x	x	x	*Chelonus inanitus*	P	[32]
**Peptides**										
Melittin	gi|58585154	Api m 4	x	-	x	x	x	*Vespula maculifrons*	T	[33]
Apamin	gi|58585166	-	x	-	x	x	x	-	-	-
Secapin	gi|58585180	-	x	-	x	x	x	*Vespa velutina nigrithorax*	U	gi|33321084
**Other toxins**										
C-type lectin	gi|328792562	-	x	-	x	-	-	-	-	-
Icarapin	gi|60115688	Api m 10	x	-	x	x	x	*Apis cerana*	T	[34]
***B.Selected trace molecules***										
**Secreted proteins**										
C1q-like protein	gi|221325614	-	x	x	x	x	x	*Nasonia vitripennis*	P	[21]
Lysozyme c-1	gi|506614822	-	x	x	x	x	-	*Ophiophagus hannah*	P+T	[20]
Peptidoglycan-recognition protein SA	gi|254910928	-	x	x	x	x	-	*Ophiophagus hannah*	P+T	[20]
Transferrin	gi|58585086	-	x	x	x	-	-	*Crotalus horridus*	T	[35]
Modular serine protease	gi|328780689	-	x	-	x	x	-	*Ophiophagus hannah*	P+T	[20]
Cathepsin F	gi|328788558	-	x	-	x	x	x	*Tityus bahiensis*	T	[28]
Cathepsin K	au9.g225.t1	-	x	-	x	x	-	*Micrurus fulvius*	T	[36]
Peptidylglycine α-hydroxylating monooxygenase	gi|328787622	-	x	x	x	x	x	*Boiga irregularis*	T	[30]
Apolipophorins	gi|571543905	-	x	x	x	x	-	*Ophiophagus hannah*	P+T	[20]
Dorsal-ventral patterning protein Sog	gi|328791019	-	x	-	x	-	-	*Ophiophagus hannah*	P+T	[20]
Laminin subunit γ-1	gi|571556732	-	x	x	x	x	x	*Ophiophagus hannah*	P+T	[20]
Vitellogenin	gi|58585104	Api m 12	x		(x)	-	x	*Bombus terrestris*	P	[37]
Glutathione S-transferase	gi|571577571	-		x	-	-	x	*Boiga irregularis*	T	[30]
Protein kinase C-binding protein NELL1	au9.g225.t1	-	x	-	-	-	x	*Ophiophagus hannah*	P+T	[20]
Odorant binding protein 14	au9.g8525.t1	-	x	-	(x)	x	-	-	-	-
Aldose 1-epimerase	gi|66541614	-	x	x	-	x	-	*Boiga irregularis*	T	[30]
poly(U)-specific endoribonuclease	gi|110760204	-	x	-	-	x	-	*Ophiophagus hannah*	P+T	[20]
Peritrophins 3-B	gi|288869483	-	x	-	-	x	-	-	-	-

### 3.3. Comparison of the Venom Composition between Honeybee Workers and Queens

In our analysis, queen bee venom was lacking six of the 34 venom toxins represented throughout all toxin categories compared to worker honeybees during winter and/or summer, in particularly group XV phospholipase A2, 5′-nucleotidase, carboxylesterase, serine protease snake, serpin 3, and c-type lectin. Next to that, two newly discovered venom toxins seemed to be solely present in queen bee venom. The first is serine proteinase stubble, which belongs to the group of proteases and peptidases. It showed 36% homology to a venom gland transcript from the diamondback rattlesnake *Crotalus adamanteus* [26]. Due to its extracellular localization together with its possible implication in regulation of the actin cytoskeleton and its role in cleavage of the extracellular matrix, it was categorized as a putative venom toxin. The other venom compound solely present in queen bees was antithrombin-III. This toxin is a serine protease inhibitor and is known for its role in the blood coagulation cascade. In insect venoms, this compound has not been found before, although it showed 31% similarity to a venom toxin in the scorpion *Tityus bahiensis* [28]. 

### 3.4. Comparison of Honeybee Venom Allergens

Among the 12 well-known honeybee venom allergens, 10 were shared by all the analyzed venoms. Api m 8, the carboxylesterase, was not present in queen venom, while it could be detected in both summer and winter worker bee venom. In insects, carboxylesterases have diverse biological functions such as metabolism of specific hormones and detoxification of dietary and environmental xenobiotics [38], although their exact function in honeybee venom has not been fully identified. 

Api m 12 or vitellogenin is a high molecular weight protein previously found in venom from *A. mellifera* and *Vespula vulgaris*, which belongs to the list of honeybee venom allergens due to its IgE-reactive allergenic properties [39]. Previously, vitellogenin could be detected in *A. mellifera ligustica* worker venom by LC-MS/MS analysis [3] and in *A. mellifera* worker venom by SDS-PAGE followed by MS/MS-based sequencing [39]. With the mass spectrometric technique we used, Api m 12 could only be found in the venom sample of queen honeybees. Due to its molecular function, which is described as lipid transporter activity and nutrient reservoir activity, we categorized vitellogenin as not a putative venom toxin, even though it is proven to be an allergen. Vitellogenin is a common yolk precursor protein in a wide range of insects [40] and was first purified from honeybee hemolymph more than 20 years ago [41]. The largest amount of vitellogenin was found in the hemolymph of honeybee queens but it was also present in workers [42,43]. Winter workers and nursing bees that remain in the hive have a high vitellogenin level in the hemolymph. The hive bee makes the transition to a foraging bee when an increase in the juvenile hormone level induces a repression of vitellogenin synthesis [44]. Honeybee vitellogenin is thought to act as a multifunctional molecule that is involved in a vast number of processes such as hormone signaling, food-related behavior, immunity, stress resistance, and longevity [45,46,47]. Recently, it was also found in honeybee venom, where it showed allergenic properties [39]. However, its role as a venom protein still remains speculative. 

## 4. Conclusions

The combination of the CPLL approach with FT-ICR MS/MS used on three different venom samples from honeybees resulted in 35 putative venom toxins, of which three had never been described in honeybee venoms before. Since the phospholipase A_2_ and melittin content in the venom were shown to follow a semestral synchronized variation, and the antigen 5-like gene was only expressed by venom gland tissue in winter bees and not in summer bees, seasonal venom variation was studied by comparing the venom proteome of summer workers with that from winter workers. Vitellogenin or Api m 12 appeared to be present in queen venom and other studies demonstrated its presence in summer worker venom, while it remains unclear whether winter bee venom contains this compound. Another intraspecific factor that causes venom variation is caste differentiation. Therefore, we compared venom from queens with worker venom, resulting in the remarkable absence of six venom toxins in queen venom. Two new venom toxins appeared to be solely present in queen venom, specifically serine proteinase stubble and antithrombin-III. The presence of several venom compounds is either seasonally restricted due to the fact that workers face different predators during winter than in summer, or is restricted in queen bees due to the fact that they need functional venom at a different time in their life than workers. Both facts lower their exposure significantly, as people are hardly stung by these honeybees. Still, they should be taken into account in the characterization of living organism response in *A. mellifera* sting. 

## Supplementary Files

Supplementary File 1
